# Damage Detection of Concrete-Filled Square Steel Tube (CFSST) Column Joints under Cyclic Loading Using Piezoceramic Transducers

**DOI:** 10.3390/s18103266

**Published:** 2018-09-28

**Authors:** Juan Zhang, Jindong Xu, Wenqiang Guan, Guofeng Du

**Affiliations:** School of Urban Construction, Yangtze University, Jingzhou 434000, China; 201671366@yangtzeu.edu.cn (J.Z.); 201771358@yangtzeu.edu.cn (J.X.); 201572319@yangtzeu.edu.cn (W.G.)

**Keywords:** concrete-filled square steel tube column (CFSSTC) joints, piezoceramic transducers, smart aggregates, cyclic loading

## Abstract

Concrete-filled square steel tube column (CFSSTC) joints are the most important parts of concrete-filled steel tube frame structures. It is of great significance to study the damage of CFSSTC joints under the seismic loads. In this paper, embedded piezoceramic transducers are used to monitor the damage of core concrete of CFSSTC joints under cyclic loading and surface-bonded piezoceramic disks are used to monitor the debonding damage of the steel tube and core concrete of two specimens. The damages of the joints under different loading levels and different loading cycles are evaluated by the received signal of the piezoceramic transducers. The experimental results show that the amplitude of the signal attenuates obviously with the appearance of damage in the joints, and the degree of attenuation increases with the development of the damage. The monitoring results from piezoceramic transducers are basically consistent with the hysteresis loops and skeleton curves of the CFSSTC joints during the cyclic loading. The effectiveness of the piezoceramic transducers are verified by the experimental results in structural health monitoring of the CFSSTC joint under cyclic loading.

## 1. Introduction

Concrete-filled steel tube structures are being widely applied in civil engineering [1,2,3,4,5]. Concrete-filled square steel tube column (CFSSTC) joints are the most important parts of concrete filled steel tube frame structures. In recent years, many scholars have performed experimental studies [6,7,8,9,10,11] and finite element analyses [12,13,14,15] of concrete-filled steel tube structures [16,17,18]. At the same time, non-destructive testing techniques, such as acoustic emission technology [19,20], ultrasonic testing technology [21], fiber optics [22] and X-ray, have been proposed for civil structures [23,24,25,26,27,28]. In recent years, piezoceramic materials have been successfully applied to structural damage monitoring or stress monitoring [29,30,31,32,33,34], including the smart aggregate enabled active sensing method [35,36,37,38,39], the piezoelectric impedance method [40,41,42,43,44] and the other methods [45,46,47,48,49]. Du et al. [50] investigated the pipeline corrosion pit detection using the time reversal technique with a piezoceramic transducer as a time reversal mirror. Gu et al. [51] studied the relationship between the change of signal received by embedded piezoceramic transducers and the early age strength growth of concrete specimens. Chalioris et al. [52] used embedded and externally bonded piezoelectric transducers to evaluate a shear-critical reinforced concrete beam. Du et al. [53] used the piezoelectric impedance method to access the structural health condition of a pipeline.

In recent years, the use of smart aggregates for damage detection and structural monitoring for structures under dynamic loads is receiving increasing attention. Xu et al. [54] proposed a new approach to damage detection of a concrete column subjected to blast loads using embedded piezoceramic smart aggregates (SAs). Fan et al. [55] used the piezoelectric-based electro-mechanical impedance (EMI) technique to evaluate damage of concrete columns under impact loads. Zhang et al. [56] used the active sensing method based on piezoelectric smart aggregates to monitor the internal damage of L-shaped concrete-filled steel tube columns under cyclic loading. The results show that the use of smart aggregate can directly and clearly reflect the damage process of the core concrete. Kong et al. [57] embedded smart aggregates in reinforced concrete bridge columns to monitor its internal damage under pseudo-dynamic loading. Liao et al. [58] used the smart aggregates to monitor the structural health of concrete column under seismic excitations. Gu et al. [59] studied the internal damage of circular reinforced concrete columns with embedded smart aggregates under seismic actions. The research results show that smart aggregates have great application potential in the health monitoring of large-volume concrete structures. Liao et al. [60] used smart aggregates to monitor the damage of reinforced concrete frame structures under earthquake excitations.

The damage research of structures under dynamic load is mainly focused on reinforced concrete structures, and there is less research on the damage of concrete-filled square steel tube column (CFSSTC) joints. The beam-column joints are the key part of the frame structures. It is important to study the damage of the structure under cyclic loading for the concrete-filled steel tube structure. Therefore, in this paper, two CFSSTC joints with installed piezoelectric transducers are fabricated. The smart aggregates are embedded in the core area of the joint and the piezoceramic disk transducer is bonded on the outer surface of the steel tube. The piezoceramically-enabled active sensing method is used to monitor the concrete damage and the steel tube debonding in the core area of the joint under low cyclic loading.

## 2. Smart Aggregate-Based Structural Health Monitoring System

**Figure 1:**
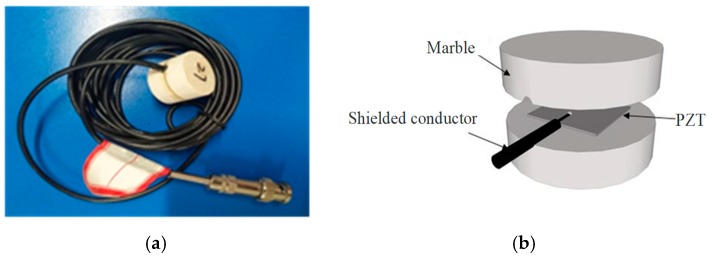
The embeddable piezoceramic smart aggregate. (**a**) The photo of a piezoceramic smart aggregate; (**b**) Three-dimensional schematic of a piezoceramic smart aggregate.

**Figure 2:**
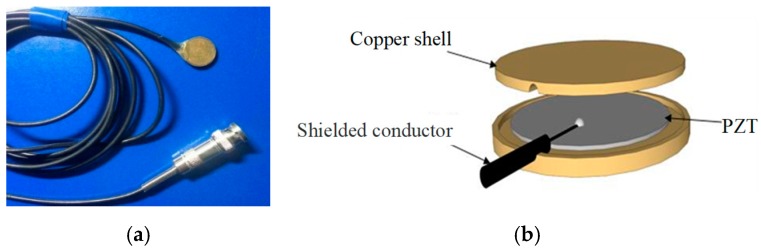
The surface bondable piezoceramic disk sensor. (**a**) The photo of piezoceramic disk; (**b**) Three-dimensional schematic of piezoceramic disk.

Lead zirconate titanate (PZT), as a type of piezoceramic material, has the advantages of good linearity, fast response, low cost, and sensing and actuating capacities [61], and has been widely used to generate and detect stress waves [62,63,64,65] for the purpose of structural damage detection [66,67,68,69] and health monitoring [70,71,72]. However, PZT is extremely fragile, and therefore it must be properly protected before deployment in civil structures. Two types of protected PZT transducers are fabricated and used in this paper. The first one is the smart aggregate, shown in Figure 1a, and formed by encapsulating a piezoelectric patch in two marble blocks, as shown in Figure 1b. With this design, the smart aggregate can be easily embedded in a concrete structure. The second type used in this paper is a piezoceramic disk transducer, as shown in Figure 2a, and it is protected by a copper shell, as shown in Figure 2b. With this design, the piezoceramic disk can be easily bonded to the surface of a structure. The detailed parameters of the selected PZT in this research are shown in Table 1.

**Figure 3:**
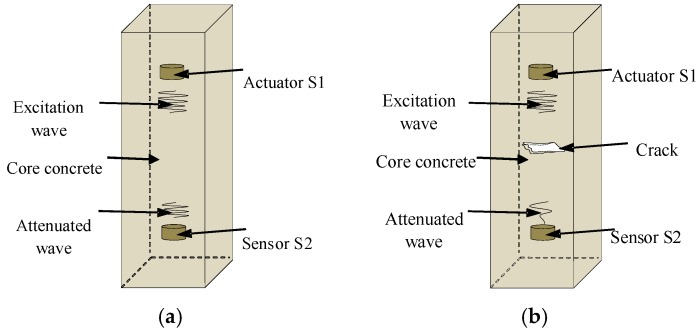
Active sensing approach to damage detection of core concrete using smart aggregates. (**a**) Core concrete in a healthy state; (**b**) Core concrete with a crack.

**Figure 4:**
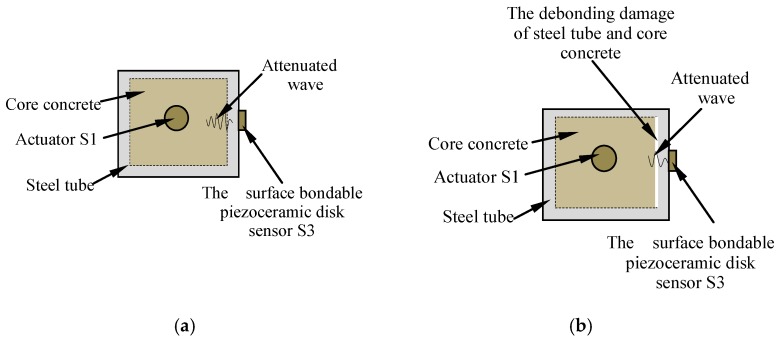
Active sensing approach to debonding detection of core concrete and steel tube. (**a**) The structure in a healthy state; (**b**) The structure under the debonding damage.

Schematic diagrams of monitoring the concrete core damages and the debonding damages based on piezoceramically-enabled active sensing approaches are presented in Figure 3 and Figure 4, respectively. Upon excitation from a signal from a function generator with amplification, the piezoelectric actuator S1 in the concrete core generates stress waves, which are transmitted to the piezoelectric sensor S2 along the concrete core of the structure. As shown in Figure 3a, when there is no damage in the concrete core, a stronger signal is received by the sensor S2. However, as shown in Figure 3b, with the presence of a crack or cracks, that function as stress relief for the stress waves, a weaker signal is detected by the sensor S2. Meanwhile, the stress waves are also transmitted along the core concrete, the interface between the concrete and steel tube, and the steel tube of the structure, and finally reach the piezoelectric sensor S3, that is bonded on the outer surface of the steel tube. As shown in Figure 4a, when there is no debonding between the concrete and the steel tube, a stronger signal is received by the sensor S3. However, as shown in Figure 4b, with the presence of a debonded area between the concrete and the steel tube, a weaker signal is detected by the sensor S3. As shown in Figure 3 and Figure 4, when the stress wave encounters cracks or debonding damage in its propagation path, the amplitude and energy of the stress wave will be attenuated. The presence of damage and the extent of damage in the specimen can be identified by an analysis of signal amplitude changes from sensors S2 and S3.

It should be noted that the energy attenuation highly depends on the severity of the cracks. Compared with other parameters such as ultrasonic velocity and the change of frequency range, the signal amplitude has better sensitivity to concrete crack damage, and the amplitude decreases with the increase of damage severity. Therefore, the signal’s amplitude can be used as the main characteristic parameter for damage monitoring. The amplitude normalization method is used to process the signals received by piezoelectric sensors to detect the damage severity. The damage index (DI) is selected as the evaluation value of the damage of the joint specimen and is defined by:

(1) $$\mathrm{DI}=\left|\frac{H_{A}-D_{A}}{H_{A}}\right|$$

where $H_{A}$ is the frequency domain amplitude of the structure in a healthy state, and $D_{A}$ is the frequency domain amplitude under the damaged status of the structure. The damage index (DI) is in the range of 0–1, which indicates the extent of the amplitude attenuation of the structure in a damage state. The larger the DI value, the more severe the damage of the structure is. When DI is 1, it means that the structure is completely damaged, and when DI is 0, it means that the structure is in the healthy state.

## 3. Experimental Setup

**Figure 5:**
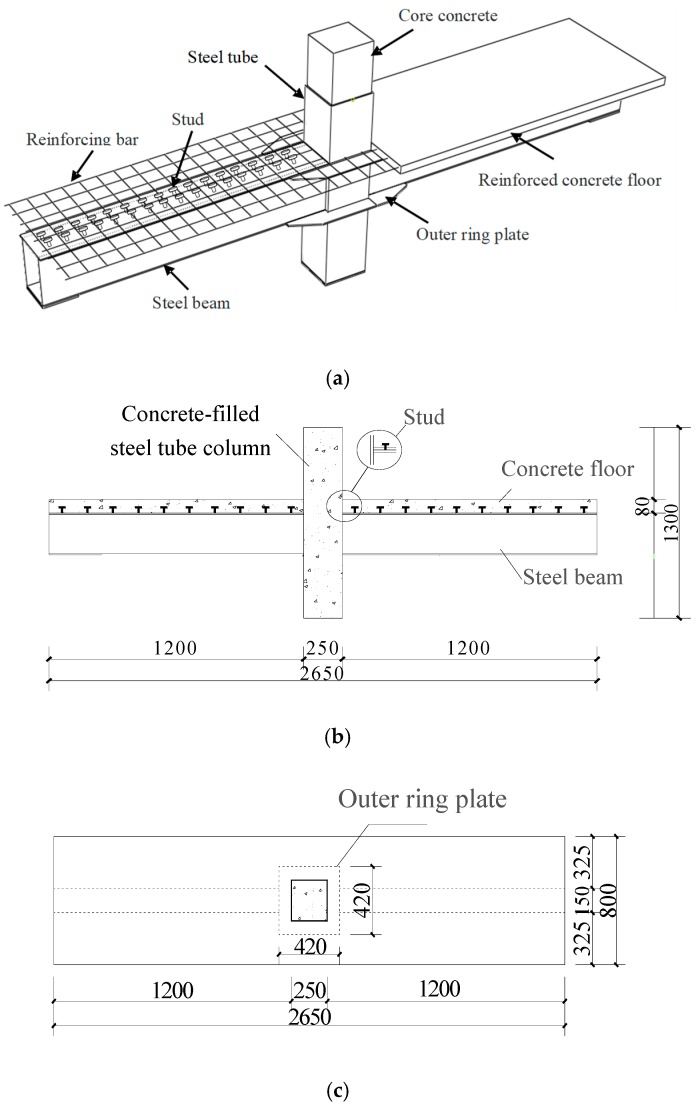
The details of the joint specimen. (**a**) Joint drawing; (**b**) The cross section drawing of joint; (**c**) The vertical view of joint.

**Figure 6:**
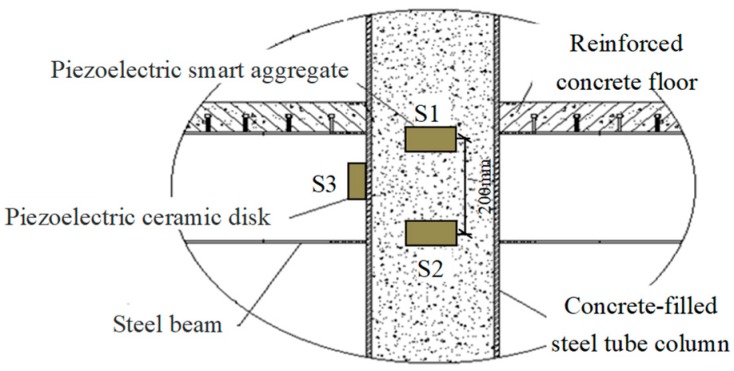
Layout of piezoceramic transducers installed in a joint.

**Figure 7:**
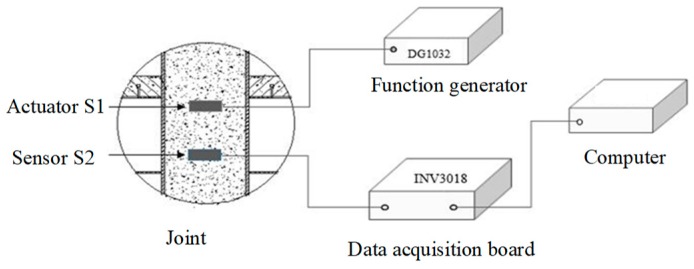
Schematic of the test monitoring system.

Two specimen joints were fabricated, named JD1 and JD2, as shown in Figure 5. A piezoelectric disk sensor was bonded the outer surface of the steel tube to identify the debonding damage of the structure in the experiment. Two SAs were embedded in structure internal to identify the internal damage of the structure. The layouts of the piezoceramic disk sensor and SAs are shown in Figure 6, and the distance between the two embedded SAs is 200 mm. S1 was used as actuator and S2 was used as sensor to monitor the internal damage of the structure. Meanwhile, S1 was used as actuator and S3 was used as sensor to identify the debonding damage of the structure. The schematic of the experimental instrumentation is shown in Figure 7. It is well known that the frequency of the signal is the main factor affecting the propagation distance of the signal in a medium. When the frequency is low, the signal can propagate a long distance in the medium. When the frequency is high, the propagation distance of the signal in the medium is greatly shortened. On the other hand, a stress wave with a higher frequency is more sensitive to smaller cracks. By considering the tradeoff between the propagating distance and sensitivity to small cracks, we choose a sine wave with the frequency of 12.35 KHz and the amplitude of 8 V as the actuator excitation signal. The signals received by sensors are collected by the INV data acquisition and analysis system, and the sampling frequency is 102.4 KHz. The data acquisition length is 5 s.

**Figure 8:**
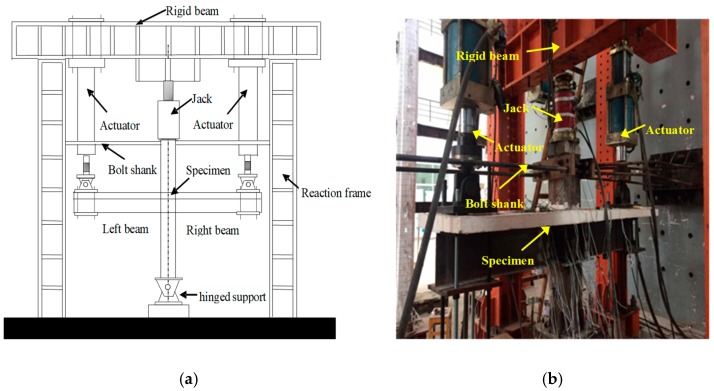
The loading setup and the specimen. (**a**) Schematic of testing apparatus and a specimen; (**b**) A photo of the testing setup and a specimen.

The pseudo-static test method is adopted in the experiment. First, the hinge support is fixed by the ground anchor bolt to avoid the overall slip during the subsequent loading process, and then the bottom end plate of the joint is fixed to the hinge support. The top of the column uses a 2000 KN jack to apply the axial force, and the jack is fixed on the rigid beam. A seismic load was applied to the beam ends on both sides using a 500 KN actuator, and the actuator was attached to the beam. Before the test, preloading along the axial direction was applied 0.15 P to stabilize the load and deformation of the specimen, and unloading. The schematic diagram of the loading device is shown in Figure 8a, and the loading device is shown in Figure 8b.

## 4. Test Loading Schedule

**Figure 9:**
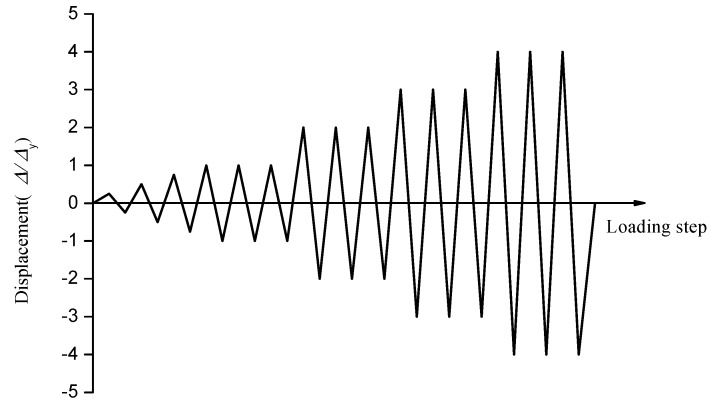
The cyclic loading schedule.

Before the test, the ABAQUS software was used to establish the joint specimen model and to load the model monotonously [73,74]. Through the analysis of the skeleton curve under the monotonic load of the joint, the yield displacement (${∆}_{y}$) and the yield load ($P_{y}$) of the joint specimens were estimated, which is used as a reference when the test is officially loaded and adjusted according to the actual situation. The displacement control loading method was used in this test. The ${∆}_{y}$ was 8 mm according to the finite element, and the single loading is performed by using 2 mm, 4 mm, 6 mm, etc. In the preloading stage, ${∆}_{y}$ was determined after the specimen has yielded. When the displacement exceeded ${∆}_{y}$, the control load was applied with a multiple of the displacement, and each loading displacement was repeated three times until the specimens damaged, and then the loading was stopped. The test loading schedule is shown in Figure 9. The amplitude of the specimen can be used as a parameter to represent the signal during the loading process. The damage of the joint specimen will cause the signal to change in amplitude, and the amplitude of the signal will continue to decay following the deepening of the damage. Therefore, the time domain signals collected by sensors under sine wave excitation are transformed into the frequency domain signal by Fourier transform. The damage of the specimen is identified, and the damage under different loading cycles with the same load is monitored according to the change of the amplitude of the signal.

## 5. Experimental Results

### 5.1. Loading History

**Figure 10:**
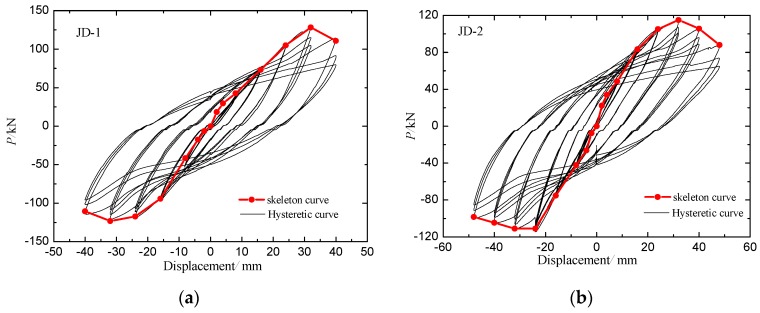
Hysteresis loops and skeleton curves of joint Specimens. (**a**) Specimen JD-1; (**b**) Specimen JD-2.

**Figure 11:**
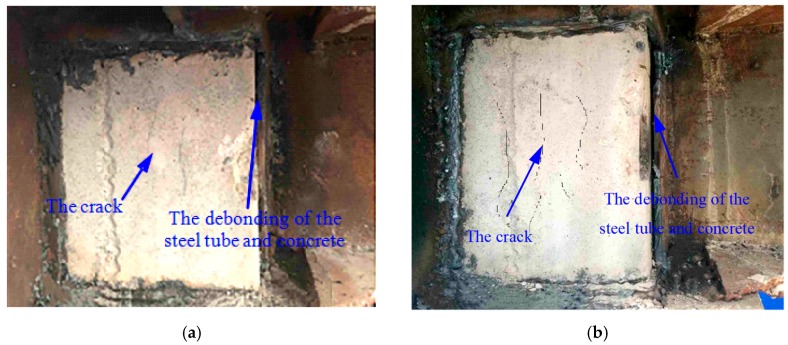
Damages to the joint specimens (cut-away view). (**a**) Specimen JD-1; (**b**) Specimen JD-2.

It must be noted that loading should be stopped if one of the following conditions is reached: (1) the weld of the steel beam in the core area of the joint is completely broken or severely buckled at the flange; (2) the lateral loading force of the specimen falls below 85% of the peak loading force. The damage phenomena of the two specimens are basically the same and JD-1 is taken as an example to describe the test phenomena. JD-1 showed the following experimental phenomena: the weld seam at the end of the beam and the external strengthened ring joint basically broke, the web plate closed to the steel tube was cracked, the concrete floor collapsed and the surface crack was basically through, the floor and the ring plate were basically separated. The hysteresis loops and skeleton curves of the two specimens are shown in Figure 10. The steel plates in the monitoring area of the specimens were peeled off after the test. It is found that the concrete inside the specimens is cracked, and the outer steel tube debonds from the inner concrete. Damage photos are shown in Figure 11.

The hysteretic loops and skeleton curves of the two specimens were analyzed, which revealed that the slope of the hysteretic loops and skeleton curves of the specimens are small at the initial stage of loading, and the loading and unloading curves of the specimens are basically coincided. Before the loading displacement is 24 mm, the specimens are in the elastic stage. Subsequently, the slope of the curves changes gradually, and the specimens enter the yielding stage. When the loading displacement is 32 mm, the load reaches the peak value, and then the load decreases with the increase of loading displacement. At the initial stage of loading, the cracking of the internal concrete is restrained because of the restraint of the external steel tube. With the increase of the loading displacement amplitude and the number of cycles, the external steel tube gradually yields, and the restraining effect of the steel tube on the inner concrete is decreasing too. The number of cracks in the concrete increases continuously, and the crack width increases continuously, which results in the gradually reduced concrete bearing capacity.

### 5.2. Damage Analysis

**Figure 12:**
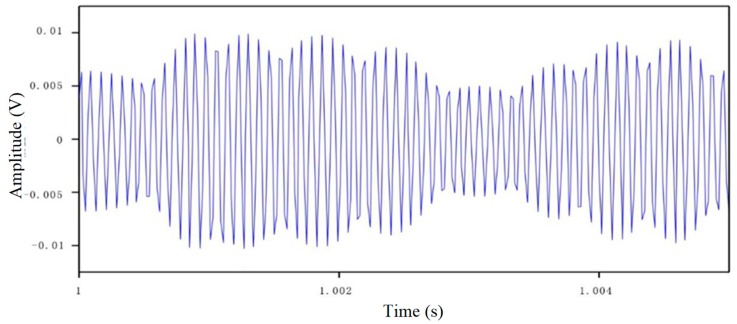
Time domain diagram of sine signals received by a smart aggregate sensor.

Figure 12 shows a time domain signal received by a smart aggregate. Though a piezoceramic sensors may experience drift for long term monitoring, we did not observe any drift in the piezoceramic transducers during all the tests for the following reasons: (1) All the tests were conducted in a relative short time frame; (2) the piezoceramic transducers were operated in the dynamic mode since they are used to generate and to detect high frequency stress waves. Given these reasons, the piezoceramic transducers in this research did not display drift.

**Figure 13:**
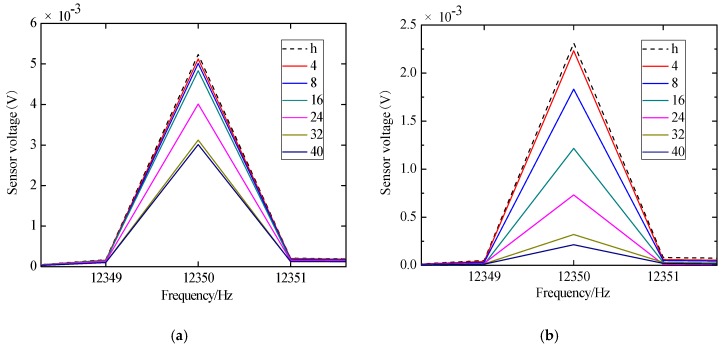
Frequency domain diagram of sine signals received by sensors of specimen JD-1. (**a**) S1-S2; (**b**) S1-S3.

**Figure 14:**
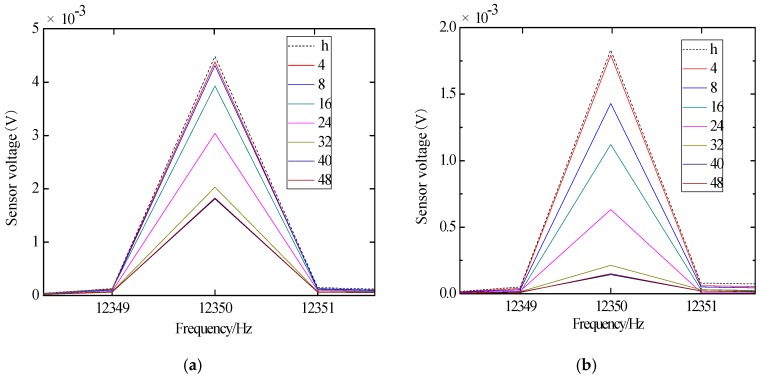
Frequency domain diagram of sine signals received by sensors of specimen JD-2. (**a**) S1-S2; (**b**) S1-S3.

In order to analyze the structural damage in the frequency domain, the time domain signal received by the sensors are subjected to a Fast Fourier Transform (FFT) to obtain the frequency domain plots. The frequency domain signals obtained by the sensors in JD-1 and JD-2 are shown in Figure 13 and Figure 14, and “S1-S2” in the figures indicates that the piezoelectric transducer S1 emits the signal and the sensor S2 receives the signal. The “h” indicates the healthy state before loading, and the remaining numbers indicate the displacement loading level. Their units are mm.

It can be concluded that the signal amplitude of the S1-S3 sensor combination is weaker than the S1-S2 one when the specimens are healthy, according to Figure 13 and Figure 14. The reason for this phenomenon is that the stress wave propagation path of sensor S1-S3 traverses two kinds of media (concrete and steel tube) and an interface between them, which leads to an attenuation of the stress waves:

#### 5.2.1. Specimen JD-1

According to Figure 13a, it can be seen that the signal amplitude attenuation is not obvious during the initial stage of displacement loading. Because of the existence of a reinforced concrete floor and the interaction between the core concrete and steel tube, the core concrete does not produce damage in the initial stage of displacement loading. Subsequently, when the loading displacement is 24 mm, the signal attenuates greatly, the reinforced concrete floor is damaged gradually, and the core concrete is cracked gradually; when the loading displacement is 32 mm, the signal amplitude attenuates to the maximum. The signal attenuation tends to be gentle due to the complete cracking of the floor and the damage of the steel beam. According to Figure 13b, it can be seen that the signal attenuates when the loading displacement is 8 mm, and the steel tube debonds from the core concrete gradually. When the loading displacement is 32 mm, the signal attenuates to the maximum, and the debonding damage is becoming more and more obvious.

#### 5.2.2. Specimen JD-2

According to Figure 14a, it can be seen that the signal amplitude attenuation is not obvious during the initial stage of displacement loading because of the existence of the reinforced concrete floor and the interaction between the core concrete and steel tube. Subsequently, when the loading displacement is 24 mm, the signal amplitude attenuates greatly. When the loading displacement is 40 mm, the signal amplitude attenuates to the maximum, and the signal attenuation gradually becomes gentle due to the complete cracking of the floor and the damage of the steel beam. According to Figure 14b, it can be seen that the signal begins to gradually decay when the loading displacement is 8 mm. The signal attenuation reaches the maximum when the loading displacement is 40 mm, and the debonding of steel tube and core concrete is obvious. The results of the signal amplitude of the specimen are basically consistent with those of the skeleton and hysteretic curves analysis.

**Figure 15:**
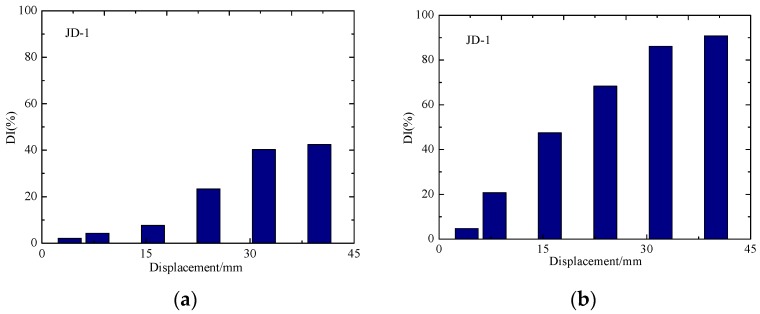
The damage indices of JD-1. (**a**) S1-S2; (**b**) S1-S3.

**Figure 16:**
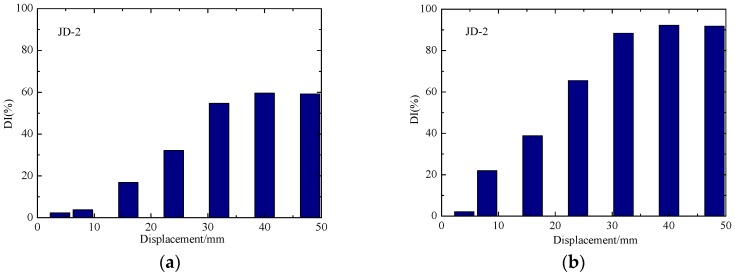
The damage indices of JD-2. (**a**) S1-S2; (**b**) S1-S3.

To carry out a more detailed and accurate analysis of the damage of the specimens, the normalized DIs of the signal amplitude of the test specimens are shown in Figure 15 and Figure 16, and the damage evolution of each specimen can be more intuitively seen from these figures. In the entire process, the DI of each sensor shows a gradual increase trend with the loading of displacement. According to Figure 15a and Figure 16a, it can be seen that the DI of concrete in the core area of the two joint is not up to 60%. As the concrete floor gradually drops out of work, the steel bars and beams in the floor bear most of the load and the damage of concrete-filled steel tube columns is smaller. The phenomenon shows that the core concrete of the joint is not damaged in large area. From the Figure 15b and Figure 16b, it can be seen that the debonding damage between concrete and steel tube occurs earlier in the core area, the DI increases linearly with the test loading, and the stress wave signal decreases sharply with the increase of the debonding damage. The DI of S1-S3 reached about 90%, indicating that the debonding damage between the core concrete and steel tube is serious.

At the end of the test, the outer wall of the steel tube in the core area of the joint specimen was removed to observe the concrete failure and the debonding damage between the core concrete and steel tube. As shown in Figure 11, the interfaces of the two joint specimens are obviously debonded, which is consistent with the result of damage identification by the S1-S3 sensors. The core concrete is basically intact, and there are some slight cracks in JD-1 and JD-2, but the interface integrity is basically maintained, which is consistent with the damage identification result of the sensors and the damage monitor results are in good agreement with the experimental results.

**Figure 17:**
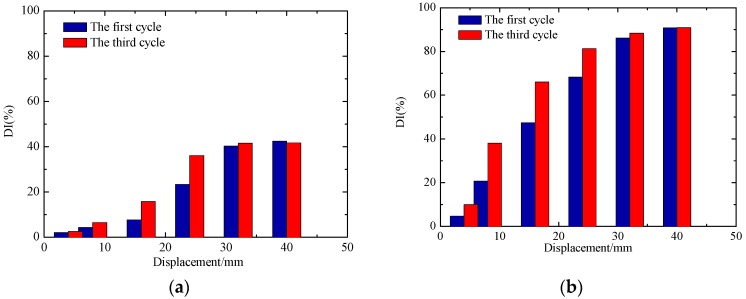
The damage indices of JD-1 in different cycles. (**a**) S1-S2; (**b**) S1-S3.

**Figure 18:**
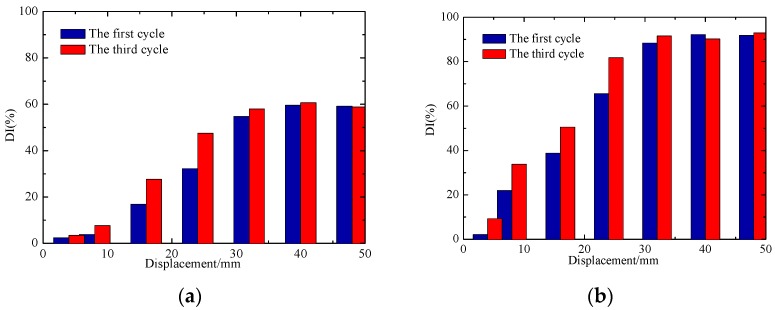
The damage indices of JD-2 in different cycles. (**a**) S1-S2; (**b**) S1-S3.

The comparisons of normalized DI for different loading cycles of JD-1 and JD-2 are respectively plotted in Figure 17 and Figure 18, which show that the DI increases gradually with the increase of the number of cycles in the same level. Though the difference between the cycles gradually increases, the overall trend is consistent at the elastic and yielding stage of the joint. In the yielding stage of the specimens, the DIs under different cycles are quite different, the reason is that the deformation of the joints develop with the increase of the load at this stage, and the attenuation of the signal amplitude increases gradually between different cycles. At the failure stage of the specimens, the load on the beam of the joint core zone is gradually weakened, and the attenuation of the signal amplitude tends to be stable between different cycles because of the damage of the steel beam.

The comparisons of normalized DI for different loading cycles of JD-1 and JD-2 are respectively plotted in Figure 17 and Figure 18, which show that the DI increases gradually with the increase of the number of cycles in the same level. Though the difference between the cycles gradually increases, the overall trend is consistent at the elastic and yielding stage of the joint. In the yielding stage of the specimens, the DIs under different cycles are quite different, the reason is that the deformation of the joints develop with the increase of the load at this stage, and the attenuation of the signal amplitude increases gradually between different cycles. At the failure stage of the specimens, the load on the beam of the joint core zone is gradually weakened, and the attenuation of the signal amplitude tends to be stable between different cycles because of the damage to the steel beam.

## 6. Conclusions

In this paper, embedded piezoceramic transducers are used to monitor the concrete core damage of concrete-filled square steel tube column (CFSSTC) joints under cyclic loading, and, in addition, a surface-bonded piezoceramic disk is used to monitor the debonding damage of the steel tube and core concrete of the specimens. The experimental results show that at the elastic stage, the attenuation of sensor signal amplitude is not obvious; at the yielding stage, the increase of load makes the deformation of joint specimen develop continuously, and the significant attenuation of the received signal amplitude indicate that the core concrete and steel tube are obviously debonded. At the failure stage of the specimens, because of the damage to the steel beam, the load on the beam of the joint core zone is gradually weakened, and the attenuation of the signal amplitude tends to be stable between different cycles. Through the analysis of the signals, the damage indices (DIs) of the core concrete of the two joints are less than 60%, which indicates that the core concrete is not completely damaged; the DIs of the debonding damage of the two joints reach about 90%, which indicates that the debonding damage of the concrete and steel tube is serious. The results of the data monitored by the smart aggregates under the low-frequency cyclic loading are basically consistent with the results of the seismic experiment of the specimens. The experimental results verify the feasibility of the use of piezoceramic transducer-enabled active sensing for structural health monitoring of CFSSTC joints. Future work will extend the piezoceramic-based structural health monitoring method developed in this paper to monitoring the internal damage monitoring of concrete-filled steel tube structures subjected to impact loading. As another task for future research, we will develop a method to distinguish between tensile or compressive damages for the concrete core in a CFSSTC.

## Figures and Tables

**Table 1:** The parameters of PZT patch.

Performance Category	Performance Value
Piezoelectric constant d_33_(10^−12^ C·N^−1^)	360
Electromechanical coupling factor (k_33_)	0.71
Density (kg/m^3^)	7600
Poisson ratio	0.35
Mechanical quality factor (Q_m_)	80
Relative permittivity ($\varepsilon_{r33}/\varepsilon_{0}$)	1800
Dielectric loss (tanδ)	0.025
Curie temperature (°C)	360

## References

[1] Varma A.H., Ricles J.M., Sause R., Lu L.W. (2004). Seismic Behavior and Design of High-Strength Square Concrete-Filled Steel Tube Beam Columns. J. Struct. Eng..

[2] Shams M., Saadeghvaziri M.A. (1997). State of the art of concrete-filled steel tubular columns. Struct. J..

[3] Shanmugam N.E., Lakshmi B. (2001). State of the art report on steel–concrete composite columns. J. Constr. Steel Res..

[4] Lee S.H., Uy B., Kim S.H., Choi Y.H., Choi S.M. (2013). Behavior of high-strength circular concrete-filled steel tubular (CFST) column under eccentric loading. J. Constr. Steel Res..

[5] Portolés J.M., Romero M.L., Bonet J.L., Filippou F.C. (2011). Experimental study of high strength concrete-filled circular tubular columns under eccentric loading. J. Constr. Steel Res..

[6] Gourley B.C., Tort C., Denavit M.D., Schiller P.H., Hajjar J.F. (2008). A Synopsis of Studies of the Monotonic and Cyclic Behavior of Concrete-Filled Steel Tube Members, Connections, and Frames.

[7] Zhang D., Gao S., Gong J. (2012). Seismic behaviour of steel beam to circular CFST column assemblies with external diaphragms. J. Constr. Steel Res..

[8] Fu G., Morita K., Ebato K. (1998). Structural behaviour of beam-to-column connection of concrete filled circular tube column and h-beam space subassemblage. J. Struct. Constr. Eng..

[9] Kang C.H., Shin K.J., Oh Y.S., Moon T.S. (2001). Hysteresis behavior of CFT column to h-beam connections with external t-stiffeners and penetrated elements. Eng. Struct..

[10] Beutel J., Thambiratnam D., Perera N. (2002). Cyclic behaviour of concrete filled steel tubular column to steel beam connections. Eng. Struct..

[11] Kim Y.J., Chae Y.S., Shin K.J., Oh Y.S., Moon T.S. Experimental results of CFT column to H-beam full-scale connections with external T-stiffeners. Proceedings of the SEWC2002.

[12] Ouyang Y., Kwan A.K.H. (2018). Finite element analysis of square concrete-filled steel tube (cfst) columns under axial compressive load. Eng. Struct..

[13] Wang Y., Li M., Fan H., Xu J.H. (2013). Finite Element Analysis on Seismic Performance of Beam-Column Joint of Concrete Filled Steel Tube Structure. Adv. Mater. Res..

[14] Du G.F., Bie X.M., Li Z., Guan W.Q. (2018). Study on constitutive model of shear performance in panel zone of connections composed of CFSSTCS and steel-concrete composite beams with external diaphragms. Eng. Struct..

[15] Han L.H., Wang W.D., Zhao X.L. (2008). Behaviour of steel beam to concrete-filled shs column frames: Finite element model and verifications. Eng. Struct..

[16] Silva A., Jiang Y., Castro J.M., Silvestre N., Monteiro R. (2017). Monotonic and cyclic flexural behaviour of square/rectangular rubberized concrete-filled steel tubes. J. Constr. Steel Res..

[17] Silva A., Jiang Y., Castro J.M., Silvestre N., Monteiro R. (2016). Experimental assessment of the flexural behaviour of circular rubberized concrete-filled steel tubes. J. Constr. Steel Res..

[18] Silva A., Jiang Y., Macedo L., Castro J.M., Monteiro R., Silvestre N. (2016). Seismic performance of composite moment-resisting frames achieved with sustainable CFST members. Front. Struct. Civ. Eng..

[19] Carpinteri A., Lacidogna G., Niccolini G. (2011). Damage analysis of reinforced concrete buildings by the acoustic emission technique. Struct. Control Health Monit..

[20] Behnia A., Chai H.K., Shiotani T. (2014). Advanced structural health monitoring of concrete structures with the aid of acoustic emission. Constr. Build. Mater..

[21] Niederleithinger E., Wang X., Herbrand M., Müller M. (2018). Processing ultrasonic data by coda wave interferometry to monitor load tests of concrete beams. Sensors.

[22] Denarie E., Saouma V.E., Iocco A., Varelas D. (2001). Concrete fracture process zone characterization with fiber optics. J. Eng. Mech..

[23] Housner G.W., Bergman L.A., Caughey T.K., Chassiakos A.G., Claus R.O., Masri S.F., Skelton R.E., Soong T.T., Spencer B.F., Yao J.T.P. (1997). Structural Control: Past, Present and Future. J. Eng. Mech..

[24] Zou F., Rao J., Aliabadi M.H. (2018). Highly accurate online characterisation of cracks in plate-like structures. NDT E Int..

[25] Herbrand M., Classen M. (2015). Shear tests on continuous prestressed concrete beams with external prestressing. Struct. Concr..

[26] Kordestani H., Xiang Y.Q., Ye X.W., Jia Y.K. (2018). Application of the random decrement technique in damage detection under moving load. Appl. Sci..

[27] Kumberg T., Schneid S., Reindl L. (2017). A wireless sensor network using gnss receivers for a short-term assessment of the modal properties of the neckartal bridge. Appl. Sci..

[28] Cao M.S., Ding Y.J., Ren W.X., Wang Q., Ragulskis M., Ding Z.C. (2017). Hierarchical wavelet-aided neural intelligent identification of structural damage in noisy conditions. Appl. Sci..

[29] Du G., Zhang J., Zhang J., Song G. (2017). Experimental study on stress monitoring of sand-filled steel tube during impact using piezoceramic smart aggregates. Sensors.

[30] Song G., Gu H., Mo Y., Mo Y.L., Hsu T.T.C., Dhonde H. (2007). Concrete structural health monitoring using embedded piezoceramic transducers. Smart Mater. Struct..

[31] Du G., Huo L., Kong Q., Song G. (2016). Damage detection of pipeline multiple cracks using piezoceramic transducers. J. Vibroeng..

[32] Wang L., Tseng K.K. (2004). Smart piezoelectric transducers for in situ health monitoring of concrete. Smart Mater. Struct..

[33] Song G., Wang C., Wang B., Song G., Wang C., Wang B. (2017). Structural health monitoring (shm) of civil structures. Appl. Sci..

[34] Du G., Li Z., Song G. (2018). A PVDF-based sensor for internal stress monitoring of a concrete-filled steel tubular (cfst) column subject to impact loads. Sensors.

[35] Xu J., Wang C., Li H., Zhang C., Hao J., Fan S. (2018). Health monitoring of bolted spherical joint connection based on active sensing technique using piezoceramic transducers. Sensors.

[36] Zhang J., Huang Y., Zheng Y. (2018). A feasibility study on timber damage detection using piezoceramic-transducer-enabled active sensing. Sensors.

[37] Yin H., Wang T., Yang D., Liu S., Shao J., Li Y. (2016). A smart washer for bolt looseness monitoring based on piezoelectric active sensing method. Appl. Sci..

[38] Xu B., Zhang T., Song G., Gu H. (2013). Active interface debonding detection of a concrete-filled steel tube with piezoelectric technologies using wavelet packet analysis. Mech. Syst. Signal. Process..

[39] Jiang T., Kong Q., Wang W., Wang W., Huo L., Song G. (2016). Monitoring of Grouting Compactness in a Post-Tensioning Tendon Duct Using Piezoceramic Transducers. Sensors.

[40] Na W., Seo D.W., Kim B.C., Park K.T. (2018). Effects of applying different resonance amplitude on the performance of the impedance-based health monitoring technique subjected to damage. Sensors.

[41] Zou F., Aliabadi M.H. (2015). A boundary element method for detection of damages and self-diagnosis of transducers using electro-mechanical impedance. Smart Mater. Struct..

[42] Shao J., Wang T., Yin H., Yang D., Li Y. (2016). Bolt looseness detection based on piezoelectric impedance frequency shift. Appl. Sci..

[43] Wang Z., Chen D., Zheng L., Huo L., Song G. (2018). Influence of axial load on electromechanical impedance (emi) of embedded piezoceramic transducers in steel fiber concrete. Sensors.

[44] Liu P., Wang W., Chen Y., Feng X., Miao L. (2017). Concrete damage diagnosis using electromechanical impedance technique. Constr. Build. Mater..

[45] Hong X., Liu Y., Liufu H., Lin P. (2018). Debonding Detection in Hidden Frame Supported Glass Curtain Walls Using the Nonlinear Ultrasonic Modulation Method with Piezoceramic Transducers. Sensors.

[46] Kaur N., Bhalla S. (2014). Combined energy harvesting and structural health monitoring potential of embedded piezo-concrete vibration sensors. J. Energy Eng..

[47] Cahill P., O’Keeffe R., Jackson N., Mathewson A., Pakrashi V. Structural Health Monitoring of Reinforced Concrete Beam Using Piezoelectric Energy Harvesting System. Proceedings of the 7th EWSHM European Workshop on Structural Health Monitoring.

[48] Cahill P., Hazra B., Karoumi R., Mathewson A., Pakrashi V. (2018). Vibration energy harvesting based monitoring of an operational bridge undergoing forced vibration and train passage. Mech. Syst. Signal Process..

[49] Quinn W., Kelly G., Barrett J. (2012). Development of an embedded wireless sensing system for the monitoring of concrete. Struct. Health Monit..

[50] Du G., Kong Q., Wu F., Ruan J., Song G. (2016). An experimental feasibility study of pipeline corrosion pit detection using a piezoceramic time reversal mirror. Smart Mater. Struct..

[51] Karayannis C.G., Chalioris C.E., Angeli G.M., Papadopoulos N.A., Favvata M.J., Providakis C.P. (2016). Experimental damage evaluation of reinforced concrete steel bars using piezoelectric sensors. Constr. Build. Mater..

[52] Chalioris C.E., Papadopoulos N.A., Angeli G.M., Karayannis C.G., Liolios A.A., Providakis C.P. (2015). Damage evaluation in shear-critical reinforced concrete beam using piezoelectric transducers as smart aggregates. Open Eng..

[53] Du G., Zhang Z. Research on evaluation index of pipeline structure damage based on piezoelectric impedance method. Proceedings of the ICPTT 2013 International Conference on Pipelines and Trenchless Technology.

[54] Xu K., Deng Q., Cai L., Ho S., Song G. (2018). Damage detection of a concrete column subject to blast loads using embedded piezoceramic transducers. Sensors.

[55] Fan S., Zhao S., Qi B., Kong Q. (2018). Damage evaluation of concrete column under impact load using a piezoelectric-based EMI technique. Sensors.

[56] Zhang J., Li Y., Du G., Song G. (2018). Damage detection of l-shaped concrete filled steel tube (L-CFST) columns under cyclic loading using embedded piezoceramic transducers. Sensors.

[57] Kong Q., Robert R., Silva P., Mo Y.L. (2016). Cyclic crack monitoring of a reinforced concrete column under simulated pseudo-dynamic loading using piezoceramic-based smart aggregates. Appl. Sci..

[58] Liao W., Wang J.X., Song G., Gu H., Olmi C., Mo Y.L. (2011). Structural health monitoring of concrete columns subjected to seismic excitations using piezoceramic-based sensors. Smart Mater. Struct..

[59] Gu H., Moslehy Y., Sanders D., Song G., Mo Y.L. (2010). Multi-functional smart aggregate-based structural health monitoring of circular reinforced concrete columns subjected to seismic excitations. Smart Mater. Struct..

[60] Liao W.I., Lin C.H., Hwang J.S., Song G. (2013). Seismic health monitoring of RC frame structures using smart aggregates. Earthq. Eng. Eng. Vib..

[61] Luo M., Li W., Hei C., Song G. (2016). Concrete infill monitoring in concrete-filled FRP tubes using a PZT-based ultrasonic time-of-flight method. Sensors.

[62] Luo M., Li W., Wang B., Fu Q., Song G. (2017). Measurement of the Length of Installed Rock Bolt Based on Stress Wave Reflection by Using a Giant Magnetostrictive (GMS) Actuator and a PZT Sensor. Sensors.

[63] Hou S., Zhang H.B., Ou J.P. (2012). A PZT-based smart aggregate for compressive seismic stress monitoring. Smart Mater. Struct..

[64] Siu S., Ji Q., Wu W., Song G., Ding Z. (2014). Stress wave communication in concrete: I. Characterization of a smart aggregate based concrete channel. Smart Mater. Struct..

[65] Siu S., Qing J., Wang K., Song G., Ding Z. (2014). Stress wave communication in concrete: II. Evaluation of low voltage concrete stress wave communications utilizing spectrally efficient modulation schemes with PZT transducers. Smart Mater. Struct..

[66] Yang Y., Hu Y., Lu Y. (2008). Sensitivity of PZT impedance sensors for damage detection of concrete structures. Sensors.

[67] Sevillano E., Sun R., Perera R. (2016). Damage detection based on power dissipation measured with PZT sensors through the combination of electro-mechanical impedances and guided waves. Sensors.

[68] Liu Y., Hu N., Xu H., Yuan W., Yan C., Li Y., Goda R., Qiu J., Ning H., Wu L. (2014). Damage evaluation based on a wave energy flow map using multiple PZT sensors. Sensors.

[69] Feng Q., Kong Q., Song G. (2016). Damage detection of concrete piles subject to typical damage types based on stress wave measurement using embedded smart aggregates transducers. Measurement.

[70] Baptista F.G., Budoya D.E., de Almeida V.A., Ulson J.A.C. (2014). An experimental study on the effect of temperature on piezoelectric sensors for impedance-based structural health monitoring. Sensors.

[71] Yang Y., Annamdas V.G.M., Wang C., Zhou Y. (2008). Application of multiplexed FBG and PZT impedance sensors for health monitoring of rocks. Sensors.

[72] Yang Y., Divsholi B.S., Soh C.K. (2010). A reusable PZT transducer for monitoring initial hydration and structural health of concrete. Sensors.

[73] Ellobody E., Young B., Lam D. (2006). Behaviour of normal and high strength concrete-filled compact steel tube circular stub columns. J. Constr. Steel Res..

[74] Classen M. (2018). Limitations on the use of partial shear connection in composite beams with steel t-sections and uniformly spaced rib shear connectors. J. Constr. Steel Res..

